# A “Detachable Polyanionic Protective Shell” Enveloping Photodynamic Cationic Nanoassemblies for Keratitis Treatment

**DOI:** 10.34133/research.1151

**Published:** 2026-02-25

**Authors:** Yuan Wei, Haoyu Zou, Yanyan Fu, Yueze Hong, Chak Kwong Cheng, Yue Wang, Meixia Zhang, Rifang Luo, Fanjun Zhang, Yunbing Wang

**Affiliations:** ^1^ National Engineering Research Center for Biomaterials, Sichuan University, Chengdu 610064, China.; ^2^ Beijing Institute of Ophthalmology, Beijing TongRen Eye Center, Beijing Key Laboratory of Ophthalmology and Visual Sciences, Beijing Tongren Hospital, Capital Medical University, Beijing, China.; ^3^ Department of Ophthalmology, and Research Laboratory of Macular Disease, West China Hospital, Sichuan University, Chengdu 610041, China.; ^4^ Department of Pharmacology, Max Planck Institute for Heart and Lung Research, Bad Nauheim, Germany.

## Abstract

Bacterial keratitis is a rapidly progressive, invasive corneal infection, necessitating the urgent development of more effective antibacterial therapies. Cationic polymers, which exert bactericidal effects by disrupting bacterial membranes, represent a promising candidate; however, their clinical application is limited by cytotoxicity associated with their positive charge. Here, we proposed a “detachable polyanionic protective shell (DPPS)” strategy to shield the positive charges of cationic polymers. Specifically, the quaternary ammonium salt (QAS)-modified polylysine was co-assembled with the photosensitizer chlorin e6 (Ce6) to obtain nanoassemblies (PQC), and then the PQC was encapsulated with polythioctic acid (PTA) to form DPPS, obtaining PQCT with functional adaptability. PQCT maintains a net negative charge under physiological conditions with good biocompatibility. In a bacterial infection environment, the reactive oxygen species (ROS) produced by Ce6 under laser irradiation will cause PTA to break bonds and degrade, DPPS to crack and be removed, the net charge of the nano-assembly to change from negative to positive, and QAS exposed to exert bactericidal functions with ROS. Both in vitro and in vivo studies demonstrated the outstanding antibacterial performance of PQCT after laser irradiation, particularly in the treatment of bacterial keratitis. This work presents a safe and effective strategy for targeted bacterial infection therapy.

## Introduction

Bacterial keratitis causes corneal disease leading to blindness [1]. The rapid progression of corneal infections, combined with the low bioavailability of conventional antibiotic eye drops, poses important challenges in clinical management [2]. Cationic polymers are a kind of antibacterial material with great clinical application value, such as polylysine, polyguanidine salt, and polyquaternary ammonium salt, etc. [3,4]. It is generally believed that the cationic polymers can disrupt the negatived bacterial cell membrane integrity, causing intracellular substance leakage [5,6]. However, the normal cytotoxicity caused by their inherent positive charge cannot be ignored either, often leading to serious consequences such as hemolysis, which greatly limits their clinical application [7–9]. Thus, strategies to mitigate the off-target toxicity of cationic polymers while preserving their antimicrobial efficacy are critically needed.

Since the cytotoxicity of cationic polymers originates from their strong positive charge, electrostatic shielding using anionic polymers presents a viable approach to mitigate toxicity. Many research have been reported [10–13]. However, these methods still weakened the antibacterial performance because the positive charge was shielded, so the antibacterial function was also shielded. We believe that these anionic polymers should be able to be removed at the appropriate time so as to enable the cationic polymer bactericidal properties to be exerted. So, in this research, we proposed a method of “detachable polyanionic protective shell (DPPS)” strategy, which covered on cationic polymers with anionic polymers to shield the positive charges. More importantly, the anionic polymers can be removed upon specific stimuli, exposing the underlying positive charge and achieving on-demand antibacterial property. To be specific, we adopted 660-nm light source as the external stimulus science near-infrared light is highly biosafe and highly operable. PTA was chosen as DPPS since it could be degraded by ROS, which could be generated by the photosensitizer under 660-nm light.

Firstly, we encapsulated the photosensitizer chlorin e6 (Ce6) within quaternary ammonium salt-modified poly-l-lysine (PLL-QAS) to form cationic nanoassemblies (PQC). Then, the PTA was adsorbed on the surface of the PQC through electrostatic interaction to form DPPS, and PQCT nanoassemblies were prepared. The PQCT exhibited a negative charge under normal physiological conditions, reducing intracellular internalization and toxicity to normal cells. When infection occurs, under the stimulation of 660-nm light stimulation, PQCT nanoassemblies generated a large amount of ROS, leading to PTA degradation and DPPS detachment. Then, the sublayer of cationic polymers will be exposed, cooperating with ROS to exert the function of on-demand antibacterial property. The excellent bactericidal performance of PQCT nanoassemblies has been verified both in vivo and in vitro, especially in the treatment of bacterial keratitis. This design of DPPS aims to balance biocompatibility and antimicrobial performance of cationic polymers, offering a promising solution for biomedical applications.

## Results and Discussion

### Preparation and characterization of PQC and PQCT nanoassemblies

**Fig. 1.**
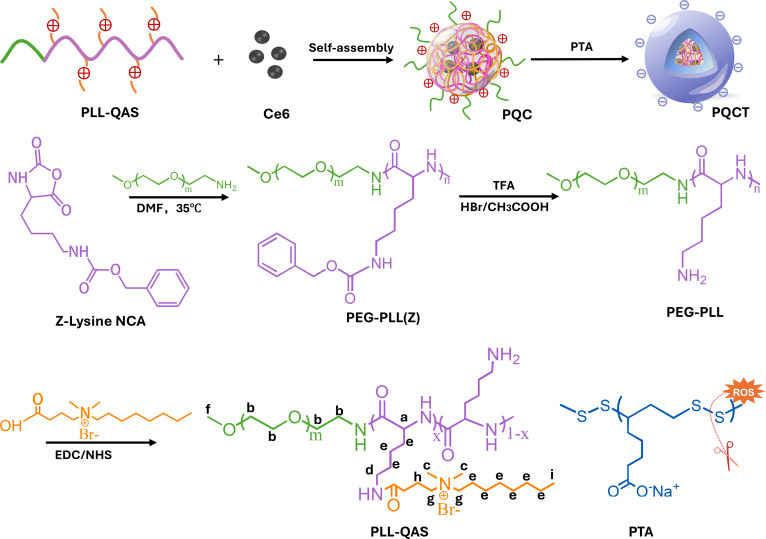
Schematic illustration of the PQCT nanoassembly preparation process.

**Fig. 2.**
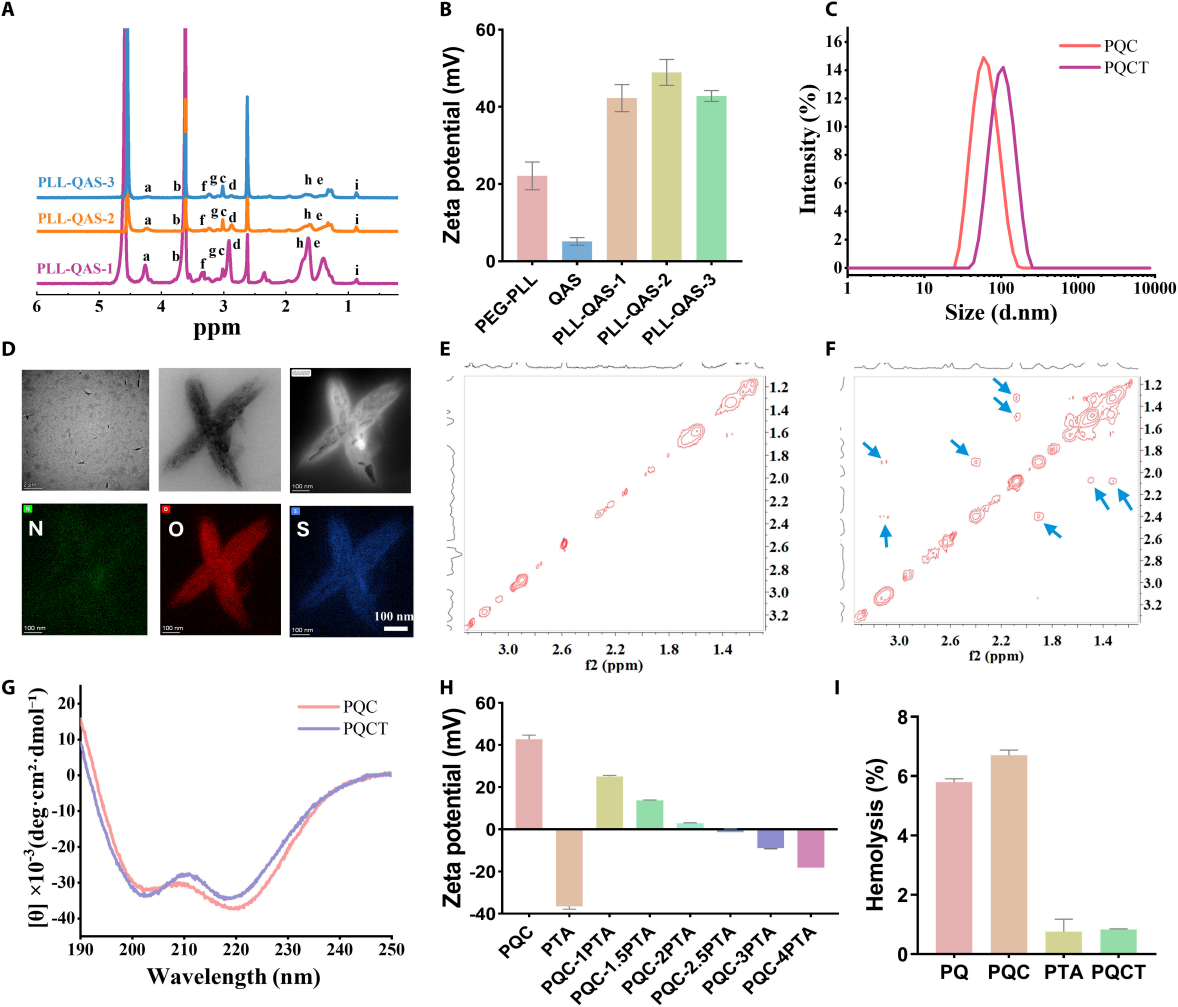
(A) ^1^H NMR spectra of PLL-QAS-1, PLL-QAS-2, and PLL-QAS-3. (B) Zeta potential of PEG-PLL, QAS, PEG-QAS-1, PLL-QAS-2, and PLL-QAS-3. (C) Size distribution of PQC and PQCT in water nanoparticles. (D) TEM image and energy-dispersive spectrometer (EDS) element map scan (N, O, and S) of PQCT nanoassemblies. (E and F) ^1^H–^1^H NOESY spectra of PQC and PQCT in D_2_O. (G) CD spectra of PQC and PQCT pure water. (H) Zeta potential of PQCT in different proportions. (I) Hemolytic ratios after the samples were incubated with 5% rabbit RBC for 2 h (*n* = 3, mean ± SD).

As shown in Fig. 1, we synthesized the cationic polymer backbone polyethylene glycol (PEG)–poly-l-lysine (PLL) through ring-opening polymerization of Lys(Z)-NCA (N-carboxy hydride). ^1^H nuclear magnetic resonance (NMR) analysis (Figs. S1 and S2) confirmed successful polymerization with a degree of polymerization (*n* = 20). We subsequently grafted QAS onto the PEG-PLL side chains at 3 different molar ratios (amino groups to QAS carboxyl groups = 1:2, 1:4, and 1:8). The ^1^H NMR spectroscopy is shown in Fig. 2A. Quantitative analysis of the ^1^H NMR spectra revealed QAS grafting efficiencies of 28.6% for PLL-QAS-2 and 61.9% for PLL-QAS-3, while PLL-QAS-1 showed insufficient signal for accurate quantification. Then, we compared the minimum antibacterial concentration (MIC) of PLL-QAS at different ratios (Table S1). As the proportion of QAS grafting increased, MIC decreased from 125 μg/ml to 31.25 μg/ml, and the best antibacterial effect was achieved at a ratio of 1:4 (PLL-QAS-2). Meanwhile, PLL-QAS-2 exhibited optimal antibacterial activity while maintaining acceptable hemocompatibility (Fig. S7). This formulation also showed the most favorable zeta potential profile (Fig. 2B). Based on these comprehensive analyses, we selected PLL-QAS-2 as the optimal candidate for subsequent preparation of Ce6-loaded PQC nanoassemblies.

As we all know, polyelectrolytes with opposite charges can self-assemble in solution through electrostatic interaction, achieving the effect of shielding charges [14]. Utilizing this principle, we first synthesized the ROS-sensitive anionic polymer PTA (Figs. S5 and S6). To generate nanoassemblies with tunable surface charges, PQC nanoassemblies were gradually introduced into PTA solutions at varying mass ratios, resulting in PQCT with different surface zeta potentials, as shown in Fig. 2H. As the PTA concentration increased, the surface charge of PQCT shifted from positive to negative. To optimize biocompatibility by minimizing cytotoxicity associated with net charge, we selected a mass ratio of 1:3 (PQC: PTA), which yielded a zeta potential of −5.54 mV for further investigation. First, the hydrodynamic size of the nanoassemblies was characterized using dynamic light scattering (Fig. 2C and Fig. S10). PQC exhibited an approximate diameter of 80 nm, whereas PQCT measured around diameter of 105 nm, attributable to PTA loading on the outer layer. Then, scanning analysis of the N, O, and S elements revealed abundant S signals on PQCT’s surface, consistent with PTA incorporation, confirming successful nano-assembly formation (Fig. 2D). The morphology of the samples was then observed using transmission electron microscopy (TEM) (Fig. 2D and Fig. S8), and it was more interesting that the sample morphology changed from spherical to spindle-shaped from PQC to PQCT. We speculate that when the negatively charged polymer was added, the electrostatic effect led to the rearrangement between molecules, which self-assembled into a spindle shape [15,16].

To elucidate the intermolecular interactions responsible for this morphological change, we performed ^1^H–^1^H nuclear overhauser effect spectroscopy (NOESY), and the results are shown in Fig. 2E and F. Only PQC was present, and no NOE signals were shown. When PTA solution was added, 4 NOE signals were observed for PQCT due to electrostatic adsorption. The spectrum displayed an intense NOE signal between the protons of the side-chain polylysines of PQC [1 to 2 parts per million (ppm)] and the PTA polymer (2.1 and 2.5 ppm), respectively. NOE signals were also present in the protons of the laterally linked QAS (3.2 ppm) and PTA polymers (2.1 and 2.5 ppm). Maybe the interaction of these functional groups is the reason for the self-assembly of PQCT. To assess the impact of charge shielding, hemolysis assays plasma assays were conducted (Fig. 2I and Fig. S12). PQC had serious hemolysis due to its positive charge, while when we wrapped PTA, the hemolysis rate was greatly reduced, indicating that our DPPS design played a great role in improving hemocompatibility and did not affect the coagulation mechanism. Additionally, considering the importance of secondary structures in cationic antimicrobial peptides, circular dichroism (CD) spectroscopy was employed to analyze conformational changes. Both PQC and PQCT displayed characteristic minima near 208 and 222 nm (Fig. 2G), indicative of α-helical structures. As α-helical peptides with amphipathic properties are known to possess enhanced antibacterial activity compared to random coils, these structural features may contribute to their efficacy [17,18].

### Charge reversal performance test

**Fig. 3.**
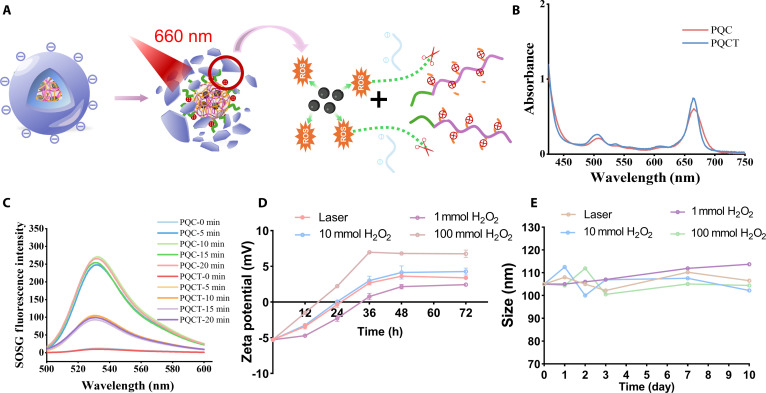
(A) Summary diagram of the charge inversion in response to ROS. (B) UV–visible spectra of PQC and PQCT. (C) SOSG fluorescence intensity curves of PQC and PQCT nanoassembly light irradiation for 0, 5, 10, 15, and 20 min (660 nm, 0.45 W cm^−2^). (D) Zeta potential and (E) size of PQCT were treated respectively with 1, 10, and 100 mM H_2_O_2_ and laser for 10 min (660 nm, 0.45 W cm^−2^).

In this work, the schematic diagram of the disintegration process of “DPPS” is shown in Fig. 3A, and its core is ROS derived from Ce6, so we characterized PQC as well as PQCT. The ultraviolet (UV) absorption spectra (Fig. 3B) revealed characteristic peaks at 400 and 660 nm for both samples, with similar intensities, indicating Ce6 successful encapsulation. To evaluate singlet oxygen (^1^O_2_) generation, we employed singlet oxygen sensor green (SOSG) fluorescent probe assays. As shown in Fig. 3C, fluorescence intensity increased progressively over irradiation time for both PQC and PQCT, and a 10-min laser irradiation was selected as the optimal condition for subsequent experiments. To demonstrate the responsive charge reversal capability of PQCT, we mixed the samples with 1, 10, and 100 mM H_2_O_2_ or laser treatment and recorded the zeta potential at different time points as well as the particle size as shown in Fig. 3D and E. After 24 h of reaction, the zeta potential in the 10 mM H_2_O_2_ group and laser-treated group approached neutral (around 0 mV) from initial values of −5.54 mV. In contrast, the 100 mM H_2_O_2_ group changed from negative to positive to +2.23 mV. After 36 h, all groups displayed positive zeta potential, indicating that ROS-triggered DPPS disassembly led to charge reversal and exposure of the QAS. At the same time, the average particle size of different samples in 1, 2, 3, 7, and 10 d was also recorded, which always fluctuated around 105 nm, reflecting good stability of PQCT nanoassemblies.

### Evaluation of biocompatibility

**Fig. 4.**
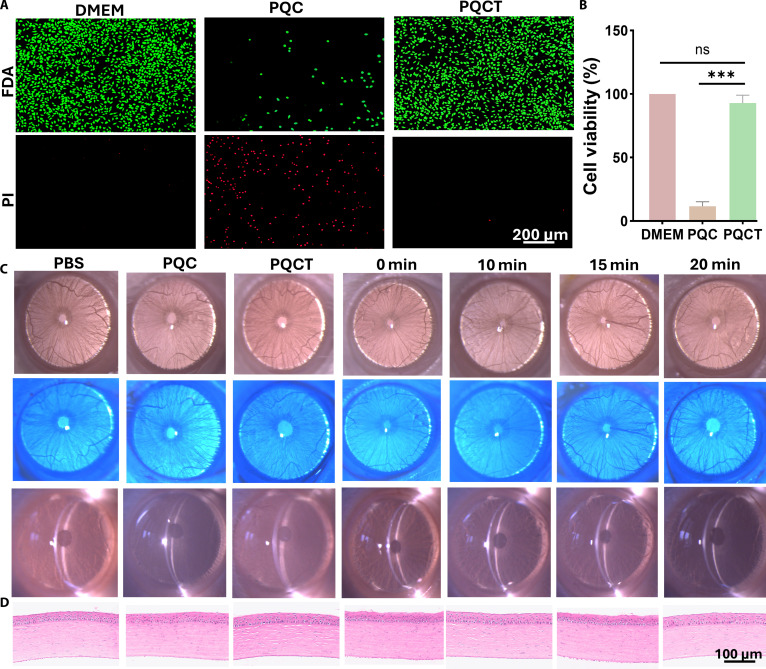
(A) Fluorescein diacetate (FDA/PI) staining image of L929 after treatment with PQC and PQCT for 24 h. (B) Cell viability was measured by CCK-8 assay (*n* = 6, mean ± SD). (C) Representative slit-lamp micrographs and normal corneal fluorescein staining after 7 d of different treatments, including daily administration of PBS, PQC, and PQCT and laser exposure for 0, 5, 10, 15, and 20 min (660 nm, 0.45 W cm^−2^). (D) H&E staining of the corneas of Sprague–Dawley rats on day 7 after different treatments.

Biocompatibility is a critical aspect of material characterization [19,20]. In this study, L929 cells were chosen to assess the cytotoxicity of the samples, as shown in Fig. 4A and B. Compared to PQC, the PQCT samples exhibited cell viability close to 100%, with cell morphology resembling that of the medium control group. This enhancement in viability can be attributed to the shielding of the positively charged surface of PQC by the polyanion layer, which prevents disruption of the negatively charged cell membrane and reduces cytotoxicity. These results demonstrated the beneficial effect of the DPPS design in improving biocompatibility. To further evaluate biocompatibility, particularly considering the eye’s sensitivity, we assessed corneal morphology, light transmittance, and microstructure in vivo. The effects of phosphate-buffered saline (PBS) solutions, PQC samples, PQCT samples, and 660-nm light exposure were studied in normal rats over a 7-d treatment period. Slit-lamp examination and corneal fluorescein staining (Fig. 4C) revealed no significant change in corneal transparency or structural integrity, with no signs of edema, congestion, excessive secretion, or increased blink frequency. On day 7, corneas from all groups were excised, fixed, and stained with hematoxylin and eosin (H&E), as shown in Fig. 4D. No significant inflammatory cell infiltration was observed in either experimental or control groups. In conclusion, the PQCT samples and the 660-nm light source developed in this study show no adverse effects on rat corneas and exhibit excellent biocompatibility.

### In vitro antibacterial assay

Bacterial infection is one of the most intractable complications in clinical practice [21,22]. Pathogens strengthen their attachment capabilities and avoid immune detection by secreting extracellular substances that facilitate biofilm formation [15,16,23]. Due to drug resistance and the formation of biofilms, conventional antibacterial agents are prone to failure [17]. Photodynamic therapy (PDT) has received increasing attention due to its spatiotemporal controllability and broad-spectrum antibacterial activity [18]. However, single PDT treatment is usually only sensitive to Gram-positive bacteria and often has an unsatisfactory bactericidal effect on Gram-negative bacteria. That is becausethe difference in the composition of the cell walls of the 2 types of bacteria [24–26]. To overcome this limitation and enhance the antibacterial activity of PDT, combination therapies such as photothermal therapy or chemotherapy are often employed. We have sufficient confidence that the ROS-mediated, PDT-synergistic cationic polymer designed in this project approaches for achieving superior antibacterial outcomes.

**Fig. 5.**
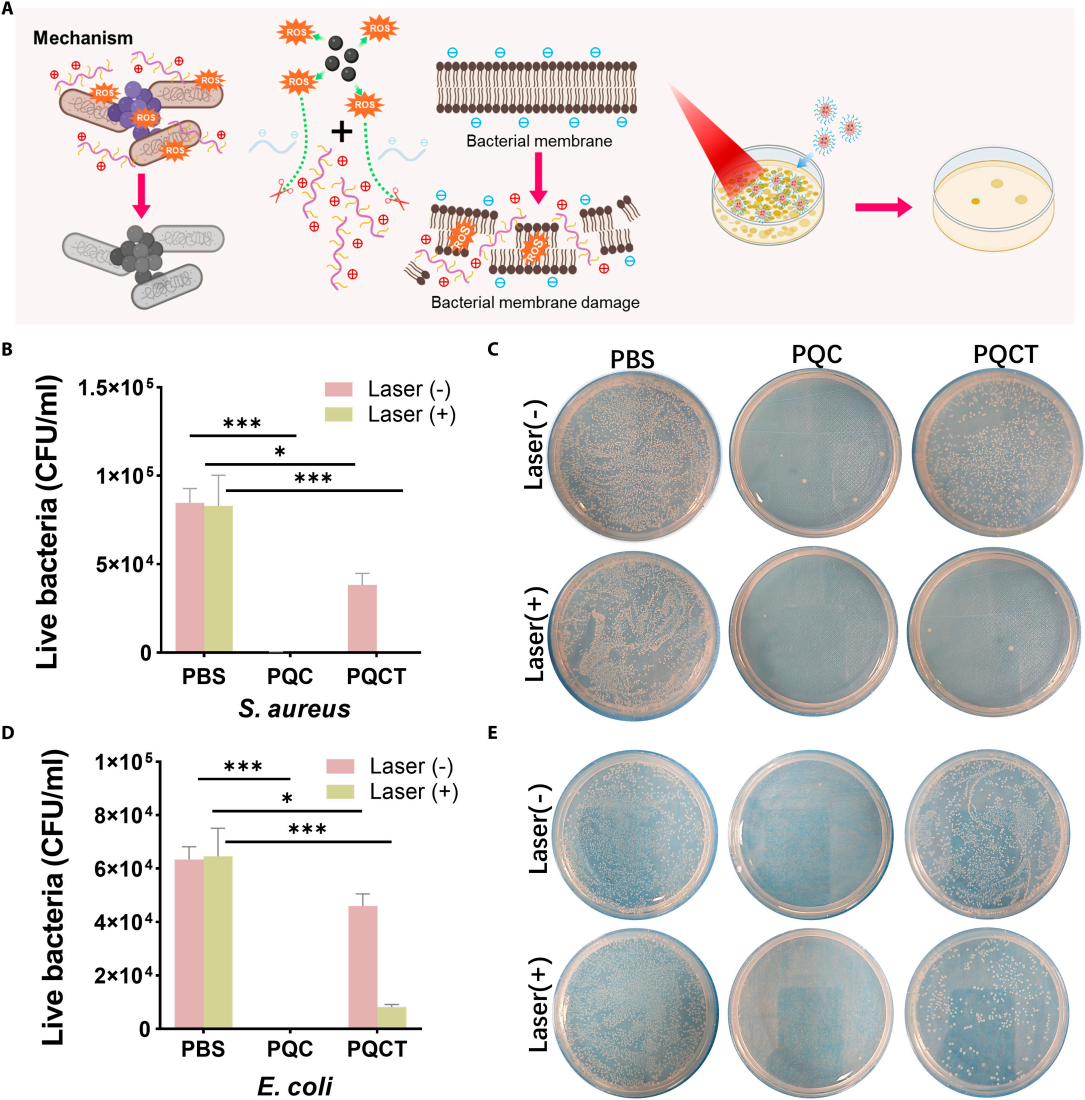
(A) Diagram of the bactericidal mechanism. (B) Quantitative analysis and (C) visual observations of *S. aureus* colonies under non-illuminated versus laser-treated conditions (10 min, 0.45 W cm^−2^). (D) Colony enumeration data and (E) photographic evidence for *E. coli* populations under identical dark and light-activated experimental setups.

**Fig. 6.**
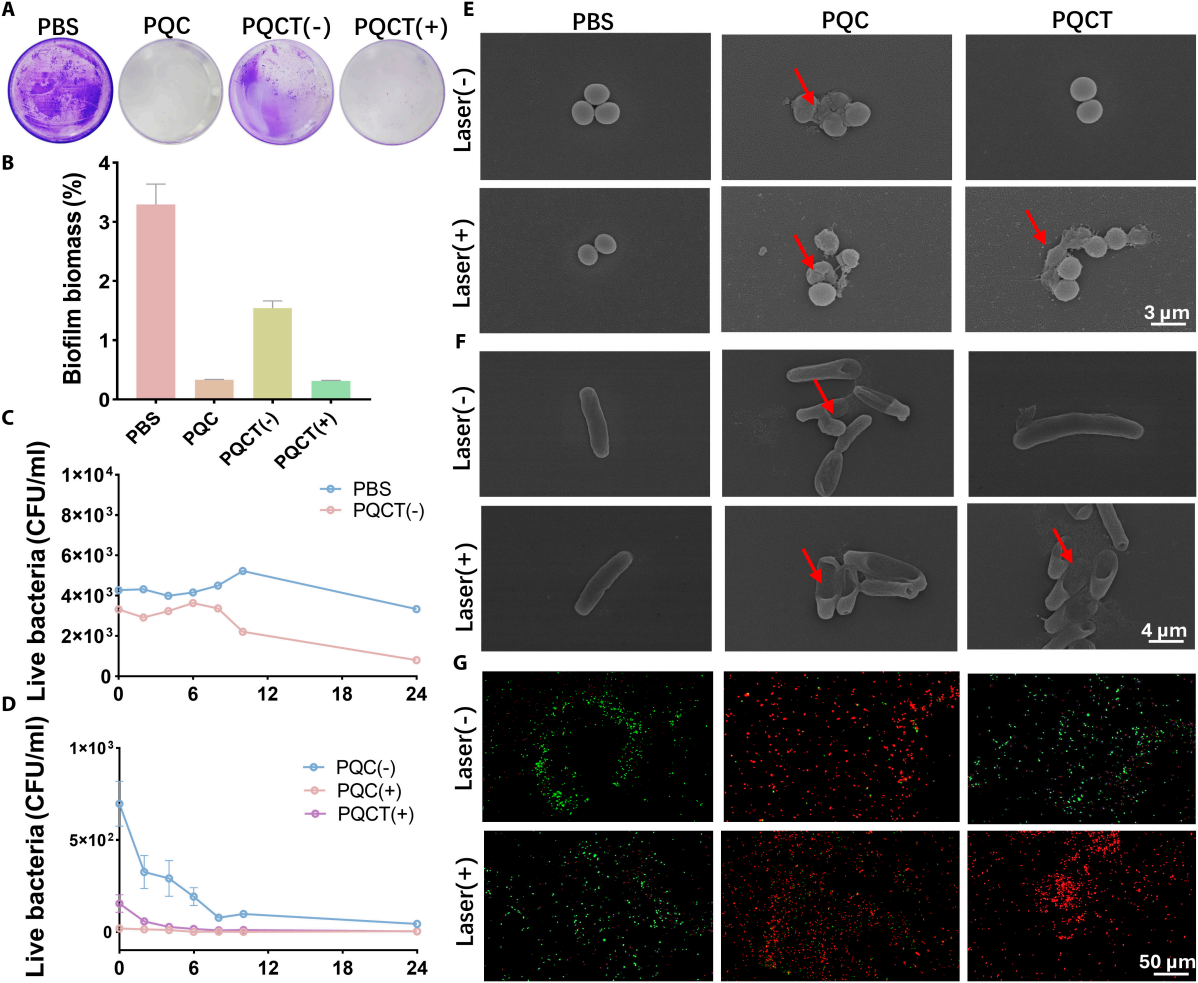
(A) Direct view of the crystal violet-stained biofilm. (B) Quantitative statistics of biofilms. (C and D) Quantitative statistics of *S. aureus* colony numbers in different sample treatments for 0, 2, 4, 6, 8, 10, and 24 h. (E and F) SEM micrographs of *S. aureus* and *E. coli* treated with different samples. (G) Laser scanning confocal microscopy images of *E. coli* treated with different samples. “(−)” means no irradiation treatment, and “(+)” means irradiation for 10 min (660 nm, 0.45 W cm^− 2^).

*Staphylococcus aureus* and *Escherichia coli* were chosen as model organisms for Gram-positive and Gram-negative bacteria, respectively, to assess the antibacterial efficacy of PQC and PQCT. The schematic diagram of the bactericidal process is shown in Fig. 5A. Three groups of samples were treated with irradiation (660 nm, 0.45 W cm^−2^) and without irradiation. The irradiated PQC demonstrated a strong positive charge, resulting in excellent bactericidal activity against both bacterial strains, with a killing rate of 99.9%. In contrast, PQCT showed similar bactericidal effects only after 10 min of irradiation, achieving killing rates of 99.9% and 87.3% against *S. aureus* and *E. coli*, respectively (Fig. 5B to E). Notably, PQCT exhibited certain antibacterial effects even in the absence of irradiation. We suspect that due to the increased ROS level in the local microenvironment at the site of bacterial infection, the “DPPS” disintegrated, exposing part of the QAS to exert antibacterial functions. To validate this hypothesis, we performed non-irradiated in vitro treatments and counted colony numbers at various time points (Fig. 6C and D and Fig. S8). Using PBS as the negative control, plate counts were performed at 2-h intervals. After 24 h of incubation, PQCT was able to kill 50% of *S. aureus* without irradiation, which aligned with our hypothesis. To further demonstrate the inhibitory effect on biofilms, we pre-added *S. aureus* suspension containing Luria–Bertani (LB) medium to the wells of a plate, incubated at 37 °C for 24 h to form a layer of biofilm. The samples were then treated, and the plate was fixed with paraformaldehyde and stained with crystal violet (Fig. 6A and B). Both the PQC (−) and PQCT (+) groups exhibited significant biofilm inhibition, with no visible purple patches. Biofilm quantity was quantified by dissolving the stained biofilms in ethanol, and the results were consistent with the observed inhibitory effects.

To clarify the bactericidal mechanisms of PQC and PQCT, morphological alterations in *E. coli* and *S. aureus* were examined through field-emission scanning electron microscopy (FESEM). After 10-min irradiation with PQC and PQCT, wrinkles and deep depressions were observed on the cell membranes of *E. coli*, while the membranes of *S. aureus* exhibited rupture and deformation, accompanied by extensive cytoplasmic leakage (Fig. 6E and F). However, in the PBS group and the PQCT (−) group, the bacteria still had smooth and regular-shaped membranes. These observations suggest that surface-exposed QAS moieties within the nanoassemblies engage with bacterial membranes via electrostatic forces, ultimately causing structural failure and cell death. The membrane-disrupting effects of PQCT were further probed through confocal laser scanning microscopy (CLSM) utilizing a dual-fluorescence staining protocol with SYTO 9 and propidium iodide (PI). Viable bacteria exhibited green fluorescence from SYTO 9 binding, while compromised cells showed red PI staining indicative of membrane integrity loss. As shown in Fig. 6G, *E. coli* bacteria in the PQC (−), PQC (+), and PQCT (+) groups displayed red fluorescence, indicating membrane rupture. However, after treatment with PBS and PQCT (+), the bacteria were mostly stained green. This further provided additional evidence that membrane integrity loss directly correlates with microbial lethality, confirming the bactericidal mechanism through cellular envelope disruption.

### In vivo therapeutic effect of PQCT nanoassemblies

**Fig. 7.**
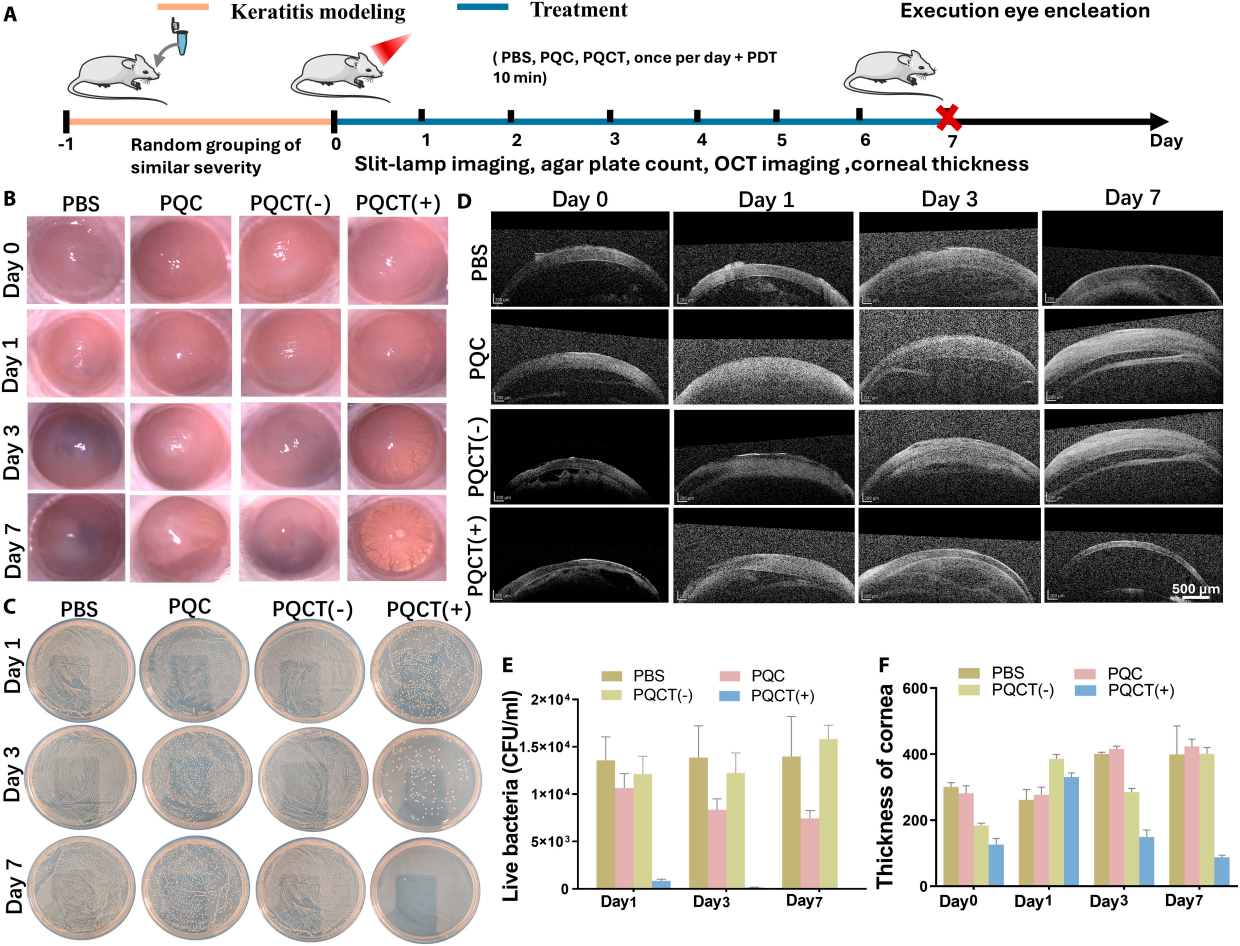
(A) Schematic representation outlining the development of keratitis animal model and therapeutic protocol. (B) Typical slit-lamp microscopy images showing *S. aureus*-induced keratitis subjected to various interventions (PBS, PQC, and PQCT at 250 μg/ml) across observation days 0, 1, 3, 5, and 7. (C) Bacterial colony visualization on agar plates following tear fluid application at 1-, 3-, and 7-d intervals. (D and F) OCT imaging of the anterior segment and quantitative analysis. (E) Matched numerical histograms illustrating bacterial counts in tear samples. The notation “(−)” indicates the absence of light irradiation, while “(+)” denotes 10-min irradiation (660 nm, 0.45 W cm^−2^).

Bacterial keratitis is mainly caused by infections of *S. aureus* and *Pseudomonas aeruginosa*, leading to irreversible damage such as corneal ulcers and perforations [27]. Encouraged by the excellent antibacterial effect in vitro, we further applied PQCT to the rat model of *S. aureus* keratitis to verify its therapeutic effect in vivo. As shown in Fig. 7A, after the establishment of the animal model of bacterial keratitis, the entire cornea was basically in an ulcerated state before the application of treatment. The infected rats were randomly divided into 5 groups and treated with PQC, PQC (+), PQCT (−), and PQCT (+). Among them, the control PBS group was not treated. On days 0, 1, 3, and 7 after the treatment, photos of each group of corneas were taken with a slit lamp to evaluate the severity and integrity of corneal infection (Fig. 7B and Fig. S13). On day 0, phenomena such as corneal opacity, edema, and congestion accompanied by purulent secretions were observed. After 1 and 3 d in the treatment group, there was no significant change in corneal ulcers in the PBS, PQC, and PQCT (−) groups, while the ulcer area in the PQCT (+) and PQC (+) groups gradually decreased to 50% and 80% of the original size, respectively. After 7 d of treatment, relatively intact corneas were clearly seen in the PQCT (+) group. The cornea in the PQC (+) group was restored to about 50% of the original, while the infections in other groups remained severe. Previously, we tested that PQC had good antibacterial ability in vitro, but the effect was not good in the treatment of keratitis in rats. We speculated that this was because after the positively charged PQC enters the ocular surface and is coated by negatively charged mucins, thereby masking its positive charge and making it less sensitive to bacteria [28–31]. In the PQC (+) group, partial corneal ulcer healing was observed, which was attributed to PDT, and ROS produced after irradiation played a role. At the same time, we also noticed that the antibacterial activity of the PQCT (−) group was inconsistent both in vitro and in vivo. This is likely because the ROS produced by local microbial infections would be gradually metabolized by the body, resulting in the inability of DPPS to be removed under no irradiation conditions. In contrast, for the PQCT (+) group, a large amount of ROS is generated locally after irradiation, causing PTA to detach, exposing the cationic polymer QAS to work synergistically in killing bacteria. As a result, the corneal inflammation heals the best. Furthermore, corneal secretions were collected to count the bacteria, as shown in Fig. 7C to E and Fig. S13. With the progress of the treatment, on day 7, almost no bacteria were detected in the PQCT (+) group, and the sterilization rate reached 99.99%. Bacterial infections and inflammatory responses usually increase the thickness of the cornea and affect its light transmittance. The central corneal thickness was observed by optical coherence tomography (OCT) imaging technology. The results were shown in Fig. 7D and F. On the 7th day, continuous and intact epithelial tissue could be seen in the PQCT (+) group, and the corneal thickness was the thinnest, indicating that the abnormal inflammatory hyperplasia was relatively mild.

**Fig. 8.**
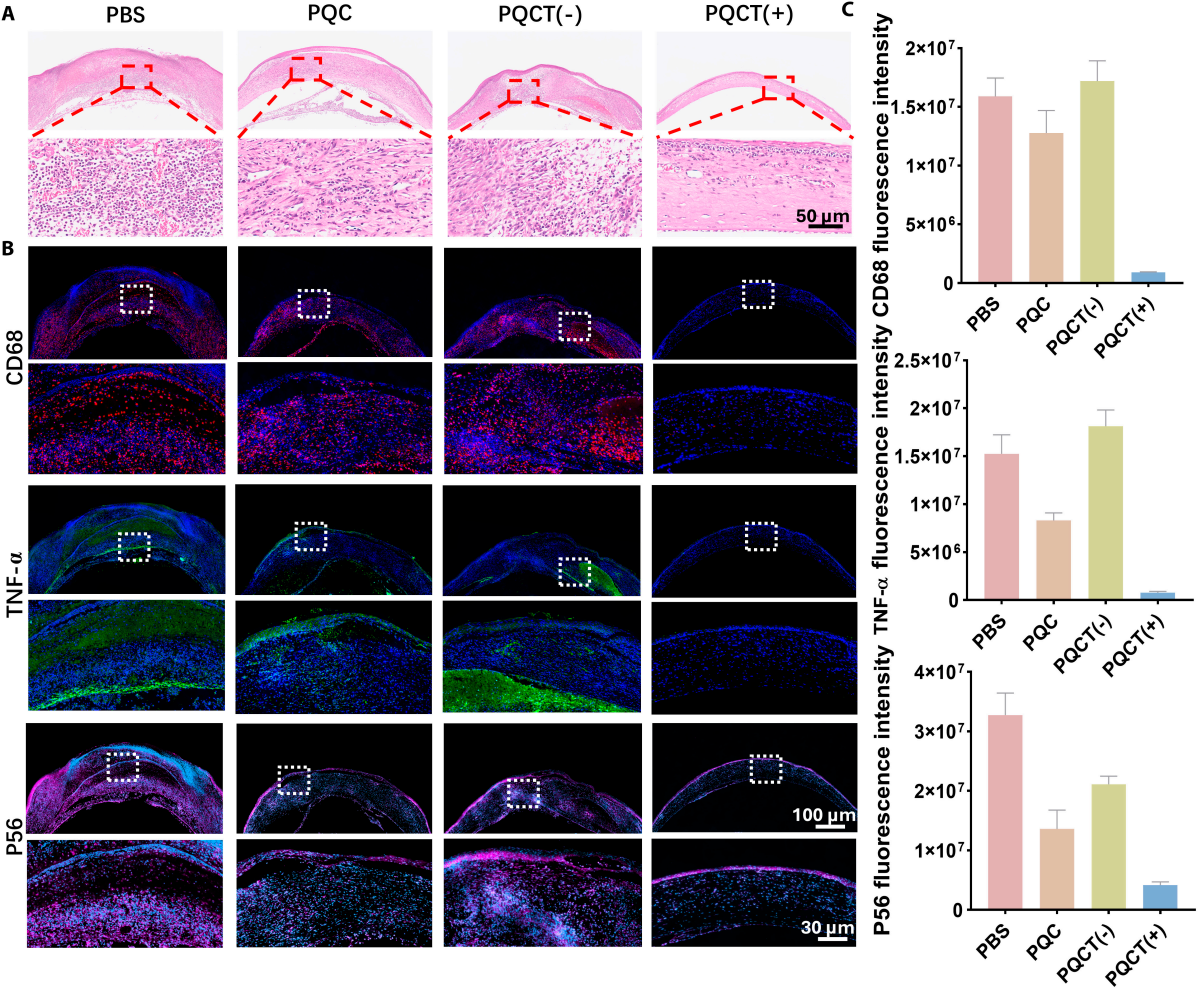
(A) H&E staining was performed on infected corneal tissues following various treatment protocols. (B) Immunofluorescence images of cytokines (CD68, TNF-α, P56) after different treatments. (C) Quantitative statistics of immunofluorescence intensity (CD68, TNF-α, P56) after different treatments.

To further evaluate the degree of corneal inflammation, the corneal tissue was removed and fixed after the 7th day of treatment and stained with H&E. The results are shown in Fig. 8A. In the PBS, PQC, and PQCT (−) control groups, there was a large infiltration of inflammatory cells and thickened tissue structure, while in the PQCT (+) group, the inflammatory cells were significantly reduced. CD68 is a representative marker of pro-inflammatory macrophages, while tumor necrosis factor-α (TNF-α) is a common pro-inflammatory cytokine. Immunofluorescence staining was performed on these 2 proteins. The results showed that the fluorescence signal of the PQCT (+) experimental group was much lower than that of the other 3 groups, indicating that the inflammatory response was the mildest and the therapeutic effect was the best, which was consistent with the results of H&E staining (Fig. 8B and C). It is well known that the nuclear factor-κB (NF-κB) signaling pathway is involved in the regulation of inflammation and immune responses in macrophages [32,33]. To understand the immune regulatory mechanism of PQCT in vivo, we labeled the NF-κB pathology-related molecule p65 protein. As expected, the expression of P56 protein was the lowest in the PQCT (+) group (Fig. 8B and C). It indicates that PQCT exerts anti-inflammatory effects on macrophages through suppression of the NF-κB signaling cascade.

## Conclusion

In this work, we designed a DPPS to shield the positive charges of cationic polymer and applied it to the treatment of bacterial keratitis. PQCT nanoassemblies exhibit ideal biocompatibility under normal physiological conditions. Upon 660-nm laser irradiation, Ce6-generated ROS triggers PTA degradation, leading to DPPS removal, the net charge of the nano-assembly to change from negative to positive, and QAS exposed to exert bactericidal functions with ROS. The excellent bactericidal performance of PQCT has been verified both in vivo and in vitro. It shows a good therapeutic effect in bacterial keratitis. Meanwhile, it inhibits the pro-inflammatory activity of macrophages and reduces the expression of inflammatory cytokines by down-regulating the NF-κB signaling pathway. In a word, PQCT is expected to be used as a new therapeutic tool for the treatment of clinical bacterial keratitis.

## Materials and Methods

### Materials and reagents

Triphosgene and trifluoroacetic acid (TFA) were sourced from McLean Biochemical Technology Co. Ltd. (Shanghai, China). mPEG-NH_2_ [molecular weight (MW), 10 kDa] was purchased from Ponsure Biotechnology Co. Ltd. (Shanghai, China). Z-lysine NCA, hydrobromic acid in acetic acid (33% w/w), 4-(dimethylamino) butyric acid, n-octyl bromide, and Ce6 were purchased from Alpha Chemical Co. Ltd. (Zhengzhou, China). Branched polyethyleneimine (PEI; MW, 25 kDa) was obtained from McLean Biochemical Technology Co. Ltd. (Shanghai, China). Tetrahydrofuran (THF), N-hydroxysuccinimide (NHS), and 1-(3-dimethyl) aminopropyl)-3-ethylcarbodiimide hydrochloride (EDC) were provided by Aladdin Biochemical Technology Co. Ltd. (Shanghai, China). Heparin sodium (Hep, 185 U/mg) and cell counting kit-8 (CCK-8) were obtained from Sigma-Aldrich (USA).

### Synthesis of PEG-PLL

PLL was synthesized following a reported procedure [34]. Briefly, Z-lysine NCA (5 g, 16.32 mmol) was dissolved in anhydrous THF (20 ml), after which mPEG-NH_2_ (molar ratio = 1:20) was added under argon protection. The reaction mixture was stirred at 37 °C for 72 h, followed by precipitation in cold ether (600 ml). The resulting white solid was isolated under vacuum, yielding 87%. Then, ε-benzyl carbamate-poly-l-lysine (PLL(Z)) was deprotected with HBr. Briefly, PEG-PLL(Z) was dissolved in TFA, and an excess of 2 times the amount of carbamate groups of hydrobromic acid/acetic acid (33% w/w) solution was added. The reaction mixture was stirred at room temperature for 6 h, after which the product was precipitated by adding a large excess of cold anhydrous ether. The precipitate was dissolved in deionized water, adjusted to neutral with 1 M sodium hydroxide solution, and subsequently dialyzed [molecular weight cutoff (MWCO), 2,000 Da] in deionized water for 72 h. The white solid PEG-PLL was obtained by freeze-drying with a yield of 70%, which was measured by ^1^H NMR spectroscopy.

### Synthesis of QAS-COOH

Dimethylaminobutyric acid hydrochloride (20 g, 11.94 mmol) was dissolved in methanol in a round-bottomed flask. To this solution, NaOH (11.46 g, 28.65 mmol) was then introduced in one portion, and the resulting mixture was stirred for 1 h. N-octyl bromide (29.95 g, 15.52 mmol) was added to the alkaline mixture under stirring. After the addition, the reaction was allowed to proceed at 65 °C for 48 to 72 h. During the reaction, the reaction termination time was determined by silica gel plate spotting. After the reaction, the unreacted monomer and the generated NaCl were removed. The specific procedure was as follows: The reaction mixture was first vacuum-filtered to remove NaOH. The filtrate was then concentrated via rotary evaporation, yielding yellow oil. This product was washed 3 times with anhydrous ether, subsequently dissolved in THF, and finally adjusted to a neutral pH using hydrochloric acid. The salt was filtered off and concentrated by rotary evaporation. The above steps were repeated until no white precipitate was produced during the THF dissolution process. The product after salt removal was precipitated with anhydrous ether 3 times and dried. The product was named QAS and characterized by ^1^H NMR spectroscopy (Figs. S3 and S4).

### Synthesis of PLL-QAS

The preparation of PLL-QAS involved carbodiimide-mediated coupling between amino and carboxyl groups through EDC·HCl/NHS activation. In standard procedures, 400 mg of PLL (45.87 μmol) was initially solubilized in 5 ml of deionized water. Separately, QAS-COOH was first dissolved in aqueous solution, followed by the addition of specific quantities of EDC·HCl and NHS to activate the carboxyl groups over a 4-h period. The activated QAS-COOH solution was subsequently combined with PLL in molar ratios corresponding to 2, 4, and 8 times the terminal amino groups of PLL. This mixture underwent continuous agitation at ambient temperature for 24 h. Post-reaction purification involved immediate dialysis against deionized water (MWCO, 2,000 Da) followed by lyophilization. These products were respectively named PLL-QAS-1, PLL-QAS-2, and PLL-QAS-3 and were determined by ^1^H NMR spectroscopy.

### Synthesis of PTA

PTA was prepared following a previously documented procedure [35]. α-Lipoic acid (2 g) was added to a round-bottom flask, ring-opening polymerization was carried out in an oil bath at 70 °C, a yellow viscous solid was obtained after stirring reaction for 4 h, and then an equal-molar NaOH solution was added and dissolved overnight. The product was dialyzed (MWCO, 500) for 3 d and lyophilized to obtain PTA, which was determined by ^1^H NMR spectroscopy.

### Hemolytic activity test of PLL-QAS

Hemolysis assay: A 10% red blood cell (RBC) suspension was prepared by diluting the stock solution with PBS. For testing, 500 μl of PBS solutions containing PEG-PLL, QAS, and PLL-QAS-1, PLL-QAS-2, and PLL-QAS-3 at 2 mg/ml concentrations were transferred into 2-ml tubes. Each polymer solution was combined with an equal volume (500 μl) of erythrocyte suspension. Negative controls consisted of blood samples mixed with pure PBS, while positive controls were prepared by lysing RBCs in deionized water. All mixtures were maintained at 37 °C for 60 min to facilitate hemolytic reactions. Post-incubation, non-lysed erythrocytes were collected through centrifugation (1,500 rpm, 15 min). Subsequently, 200-μl aliquots of the clarified supernatants were pipetted into a 96-well microplate for spectrophotometric analysis. Absorbance values at 540 nm were recorded using a BioTek Synergy H1 microplate reader. The percentage hemolysis was determined through the following computational formula:

$$\text{Hemolysis}\left(\%\right)=\frac{\left(\text{ODsamples}-\text{ODnegative}\right)}{\left(\text{ODpostive}-\text{ODnegative}\right)}\times 100$$ (1)

### MIC assays of PLL-QAS

To simulate physiological condition well, *S. aureus* was selected to investigate minimum inhibitory concentration (MIC) in LB medium. The concentration of bacteria in LB medium was 2 × 10^6^ colony-forming units (CFU)/ml. The double dilution method was used for measurement. The samples PEG-PLL, QAS, PLL-QAS-1, PLL-QAS-2, and PLL-QAS-3 were progressively diluted in 96-well plates from 1 mg/ml to 7.8 μg/ml and incubated for 6 h at 37 °C. The bacterial survival rates were quantified using standardized calculation methods.

### Preparation and characterization of PQC and PQCT nanoassemblies

The PQC was synthesized via a dialysis-based approach. In brief, a mixture containing Ce6 (8 mg) and PLL-QAS (32 mg) dissolved in 2 ml of dimethyl sulfoxide (DMSO) was gradually introduced into 18 ml of deionized water under continuous agitation, followed by transfer into dialysis tubing (MWCO, 1,000) for 72 h. PQCT was similarly prepared. PLL-QAS (0.5 mg/ml) was added dropwise into PTA solutions (0.5, 1.0, 1.5, and 2.0 mg/ml) dissolved in deionized water under stirring. Hydrodynamic diameter measurements and surface charge analysis of PQC and various PQCT formulations were conducted using a Malvern Zeta-sizer Nano ZS instrument. Morphological characteristics and nanoparticle dimensions were examined through field-emission TEM (JEM-100CXII, 100 kV). Optical absorption profiles were recorded using a Shimadzu UV2600 spectrophotometer. Two-dimensional NOESY ^1^H NMR spectra were acquired on a JEOL AVANCE III HD 400 MHz spectrometer, employing a spectral width of 4,000 Hz with a collection of 1,024 data points.

### In vitro singlet oxygen (^1^O_2_) generation detection

A 5 mM stock solution of SOSG was prepared by mixing 100 μg of SOSG with 33 μl of methanol. Prior to experimentation, this stock solution was adjusted to a 10 μM concentration through dilution. The water-compatible SOSG solution was then utilized for testing. Circular samples (10 mm diameter) were positioned in 48-well plates containing 500 μl of deionized water. Laser irradiation (660-nm wavelength, 0.45 W cm^−2^) was applied for varying durations (0, 5, 10, 15, and 20 min). Subsequent fluorescence measurements were conducted using a Hitachi F-7000 fluorescence spectrophotometer (Japan) with excitation at 480 nm and emission scanning from 500 to 650 nm.

### Hemolytic activity test of PQC and PQCT

The RBC suspension was adjusted to a 10% concentration using normal saline. In 2-ml microcentrifuge tubes, 500-μl aliquots of normal saline containing PQ (PLL-QAS), PQC, PTA, and PQCT at 2 mg/ml concentrations were prepared, followed by the addition of an equal volume (500 μl) of the blood suspension. After incubation at 37 °C for 2 h, the hemolysis rate was calculated.

### ROS triggered the changes in the potential and size of nanoassemblies

A 500 μg/ml solution of PQCT nanoassemblies was prepared and divided into experimental groups containing 1, 10, and 100 mM H_2_O_2_ concentrations. An additional group received light irradiation treatment (660 nm, 0.45 W cm^−2^) for 10 min. Zeta potential measurements were conducted at 0-, 12-, 24-, 36-, 48-, and 72-h intervals, while nanoparticle size analysis was performed at 0-, 1-, 2-, 3-, 7-, and 10-d time points.

### In vitro cytotoxicity assay

Mouse fibroblast (L929) cell was used as a mammalian cell model to test the cytotoxicity of the complex. These cells were maintained in Dulbecco’s modified Eagle’s medium (DMEM) enriched with 10% fetal bovine serum and 1% penicillin/streptomycin solution under standard culture conditions (37 °C, 5% CO_2_ atmosphere). Upon reaching 80% confluency, cells were detached using trypsinization and subsequently plated into 96-well microplates at a density of 1 × 10^4^ cells per well, followed by 24-h incubation. Experimental groups were exposed to 500 μg/ml concentrations of PQC and PQCT dissolved in physiological saline solution for 24 h, with untreated culture medium serving as the positive control group. Cellular viability was quantitatively measured after the treatment period using CCK-8 colorimetric assay.

Live/dead cell testing: Following 24-h treatments, L929 cultures were dual-labeled with fluorescein diacetate (green fluorescence) and PI (red fluorescence) for live/dead differentiation. Cellular morphology and fluorescence patterns were documented using a NexCore NIB900 inverted fluorescence microscope (USA).

### In vivo biocompatibility assay

The experimental design involved 8 distinct groups subjected to PBS, PQC, PQCT, and light-based interventions. Each treatment arm contained no fewer than 3 rats. The eye drops of PBS, PQC, and PQCT nanoassemblies were given to rats every day. The laser irradiation time of the light treatment group was 0, 5, 10, 15, and 20 min (660 nm, 0.45 W cm^−2^). Post-treatment evaluation on day 7 included slit-lamp biomicroscopy followed by euthanasia procedures. Excised corneal tissues underwent histological processing for H&E staining analysis.

### In vitro antibacterial assay

To assess the antibacterial properties of PQC and PQCT, *S. aureus* (Gram-positive) and *E. coli* (Gram-negative) were selected as model organisms. Initial bacterial cultures were grown in LB medium at 37 °C for 24 h, achieving a standardized concentration of 1 × 10^8^ CFU/ml. The *S. aureus* suspension was then diluted to 2 × 10^6^ CFU/ml using PBS buffer. Experimental mixtures containing 150 μl of either 250 μg/ml PQC or PQCT solutions combined with 50 μl of bacterial suspension were transferred into a 48-well plate, with PBS serving as negative control. Separate treatment groups received either no light exposure or 660-nm light (0.45 W cm^−2^) for 10 min. Following 6-h incubation at 37 °C, 800 μl of PBS-diluted bacterial suspensions was prepared for subsequent analysis. Microbial viability was determined through agar plate culturing and colony enumeration. For *E. coli* testing, identical procedures were implemented, except the concentration of the samples was adjusted to 500 μg/ml.

The antibacterial mechanism was examined using scanning electron microscopy (SEM). Silicon substrates were submerged in the prepared bacterial suspension for 6 h, treated with 2.5% glutaraldehyde for 24-h fixation, and subsequently subjected to sequential dehydration using ethanol/water mixtures with increasing concentrations (30, 50, 70, 90, and 100 wt %).

Live/dead test: The bacterial cultures were initially grown in LB broth at 37 °C for 24 h to achieve 1 × 10^8^ CFU/ml, followed by dilution of *S. aureus* to 2 × 10^7^ CFU/ml using PBS. Test solutions (250 μl each of PQC and PQCT at 250 μg/ml) were combined with equal volumes of bacterial suspension in 48-well plates. Following a 6-h exposure at 37 °C, bacterial pellets were collected through centrifugation (3,500 rpm, 15 min), reconstituted in 1 ml of PBS, and stained with 1.5 μl of SYTO 9 combined with 1.5 μl of PI under dark conditions for 15 min. After repeating the centrifugation process to remove residual dyes, the bacterial samples were finally resuspended in fresh PBS for CLSM (Leica TCS SP5, Germany) observation.

ROS-responsive bactericidal experiment: First, the bacteria were grown in LB broth at 37 °C for 12 h to achieve a final concentration of 1 × 10^8^ CFU/ml. Subsequently, *S. aureus* suspensions were adjusted to 2 × 10^6^ CFU/ml using PBS. In a 48-well culture plate, 150-μl aliquots of PQC and PQCT solutions (250 μg/ml concentration) were combined with 50 μl of bacterial suspensions, employing PBS as experimental control. Test groups underwent either dark conditions or light exposure (660 nm, 0.45 W cm^−2^ intensity) for 10 min. After incubation at 37 °C, colony-coating plates were counted every 2 h.

Inhibition of biofilm experiments: First, the bacteria were grown in LB broth at 37 °C for 24 h to achieve a concentration of 1 × 10^8^ CFU/ml. Subsequently, *S. aureus* suspensions were adjusted to 2 × 10^7^ CFU/ml using fresh LB medium. One milliliter of bacterial solution and 1 ml of sample were added to a 12-well plate and incubated at 37 °C overnight, and the medium was absorbed and dried in a super-clean table for 10 min. Biofilms were immobilized through 15-min paraformaldehyde treatment and subsequently colored with crystal violet solution for an equivalent duration. Following PBS rinsing to remove excess stain, biofilm visualization was performed. For quantitative analysis, 500 μl of ethanol was employed to solubilize bound crystal violet, with biofilm biomass assessed by measuring optical density at 600 nm.

### In vivo infected keratitis assay

Female Sprague–Dawley rats aged 8 weeks, with body weights around 200 g, were chosen for in vivo anti-infection studies. Surgical interventions were conducted following a 1-week acclimatization period. The experimental protocol involving vaginal irritation assessments received approval from the Ethics Committee of the Tianjin customs district industrial products safety and technical center (authorization ID: 2300029-3). Corneal infection models were established by administering 20 μl of *S. aureus* bacterial suspension (10^7^ CFU/ml) onto abraded ocular surfaces in Sprague–Dawley rats. Twenty-four hours post-inoculation, subjects demonstrating comparable infection severity were randomly allocated into 5 treatment groups (*n* = 12 each): PBS control, PQC, and PQCT (250 μg/ml). Except for PBS, the PQC and PQCT groups of the Sprague–Dawley rats were irradiated under laser (660 nm, 0.45 W cm^−2^) for 10 min. Throughout the 7-d therapeutic regimen, the periocular tissue was sampled with a cotton swab, immersed in 1 ml of PBS solution. The specimens were treated with ultrasonic solution for 1 min, followed by microbial colony quantification using agar plate cultures at 24-h, 72-h, and 7-d intervals. Corneal thickness was measured, and the corneas were observed under slit lamp on days 0, 1, 3, and 7. Following 7 d of observation, excised corneal tissues underwent fixation in 4% paraformaldehyde solution, sequential dehydration through ethanol gradients, paraffin embedding, and sectioning. Tissue sections underwent histological analysis through H&E staining and were further processed for immunofluorescence studies targeting inflammatory markers (TNF-α, CD68, and P65) to assess immune cell infiltration and inflammatory pathway activation.

### Statistical analysis

Unless specified, experiments described in this chapter were performed separately with a minimum of 3 replicates. Data processing was conducted using SPSS11.5 software, with results presented as mean ± SD. Group comparisons for statistical significance were evaluated through one-way analysis of variance (ANOVA) analysis. A threshold of *P* < 0.05 was established to denote significance levels (*P < 0.05, **P < 0.01).

## Data Availability

The data are freely available upon request.

## References

[1] Tuft S, Somerville TF, Li J-PO, Neal T, De S, Horsburgh MJ, Fothergill JL, Foulkes D, Kaye S. Bacterial keratitis: Identifying the areas of clinical uncertainty. Prog Retin Eye Res. 2022;89: Article 101031. 34915112 10.1016/j.preteyeres.2021.101031

[2] Teirlinck E, Xiong R, Brans T, Forier K, Fraire J, Van Acker H, Matthijs N, De Rycke R, De Smedt SC, Coenye T, et al. Laser-induced vapour nanobubbles improve drug diffusion and efficiency in bacterial biofilms. Nat Commun. 2018;9(1):4518. 30375378 10.1038/s41467-018-06884-w PMC6207769

[3] Jiao Y, Niu L-N, Ma S, Li J, Tay FR, Chen J-H. Quaternary ammonium-based biomedical materials: State-of-the-art, toxicological aspects and antimicrobial resistance. Prog Polym Sci. 2017;71:53–90. 32287485 10.1016/j.progpolymsci.2017.03.001 PMC7111226

[4] Rodrigues de Almeida N, Han Y, Perez J, Kirkpatrick S, Wang Y, Sheridan MC. Design, synthesis, and nanostructure-dependent antibacterial activity of cationic peptide amphiphiles. ACS Appl Mater Interfaces. 2018;11(3):2790–2801. 10.1021/acsami.8b17808 PMC7199185 30588791

[5] Bacalum M, Radu M. Cationic antimicrobial peptides cytotoxicity on mammalian cells: An analysis using therapeutic index integrative concept. Int J Pept Res Ther. 2014;21(1):47–55.

[6] Paterson DJ, Tassieri M, Reboud J, Wilson R, Cooper JM. Lipid topology and electrostatic interactions underpin lytic activity of linear cationic antimicrobial peptides in membranes. Proc Natl Acad Sci USA. 2017;114(40):E8324–E8332. 28931578 10.1073/pnas.1704489114 PMC5635876

[7] Gong C, Sun J, Xiao Y, Qu X, Lang M. Synthetic mimics of antimicrobial peptides for the targeted therapy of multidrug-resistant bacterial infection. Adv Healthc Mater. 2021;10(22):2101244. 10.1002/adhm.202101244 34410043

[8] Zhang B, Lu D, Wang DBR, Kok ZY, Chan-Park MB, Duan H. Enzyme-responsive polyion complex nanoparticles of cationic antimicrobials for activatable antibacterial therapy. Adv Funct Mater. 2024;34(46):2407869.

[9] Fjell CD, Hiss JA, Hancock REW, Schneider G. Designing antimicrobial peptides: Form follows function. Nat Rev Drug Discov. 2012;11(1):37–51. 10.1038/nrd3591 22173434

[10] Fan X-L, Hu M, Qin Z-H, Wang J, Chen X-C, Lei W-X, Ye W-Y, Jin Q, Ren K-F, Ji J. Bactericidal and hemocompatible coating via the mixed-charged copolymer. ACS Appl Mater Interfaces. 2018;10(12):10428–10436. 29508992 10.1021/acsami.7b18889

[11] Yu H, Liu L, Yang H, Zhou R, Che C, Li X, Li C, Luan S, Yin J, Shi H. Water-insoluble polymeric guanidine derivative and application in the preparation of antibacterial coating of catheter. ACS Appl Mater Interfaces. 2018;10(45):39257–39267. 30346131 10.1021/acsami.8b13868

[12] Hoque J, Ghosh S, Paramanandham K, Haldar J. Charge-switchable polymeric coating kills bacteria and prevents biofilm formation in vivo. ACS Appl Mater Interfaces. 2019;11(42):39150–39162. 31550124 10.1021/acsami.9b11453

[13] Tan J, Fang Y, Yang C, Tay J, Tan N, Krishnan NDOB, Chua BL, Zhao Y, Chen Y, Hedrick JL, et al. pH-responsive polymeric micelle dynamic complexes for selective killing of Helicobacter pylori. Biomacromolecules. 2023;24(12):5551–5562. 37828909 10.1021/acs.biomac.2c01374

[14] Lappan U, Naas C, Scheler U. Influence of the mixing ratio on the dynamics of polymer segments in polyelectrolyte complexes. Macromol Chem Phys. 2021;222(7):2000445.

[15] Carniello V, Peterson BW, van der Mei HC, Busscher HJ. Physico-chemistry from initial bacterial adhesion to surface-programmed biofilm growth. Adv Colloid Interf Sci. 2018;261:1–14. 10.1016/j.cis.2018.10.005 30376953

[16] Li W, Xiao X, Qi Y, Lin X, Hu H, Shi M, Zhou M, Jiang W, Liu L, Chen K, et al. Host-defense-peptide-mimicking β-peptide polymer acting as a dual-modal antibacterial agent by interfering quorum sensing and killing individual bacteria simultaneously. Research. 2023;6:0051. 36930779 10.34133/research.0051 PMC10014070

[17] Zhang F, Hu Q, Wei Y, Meng W, Wang R, Liu J, Nie Y, Luo R, Wang Y, Shen B. Surface modification of titanium implants by pH-responsive coating designed for self-adaptive antibacterial and promoted osseointegration. Chem Eng J. 2022;435: Article 134802.

[18] Liu C, Cao Y, Cheng Y, Wang D, Xu T, Su L, Zhang X, Dong H. An open source and reduce expenditure ROS generation strategy for chemodynamic/photodynamic synergistic therapy. Nat Commun. 2020;11(1):1735. 32269223 10.1038/s41467-020-15591-4 PMC7142144

[19] Williams DF. The plasticity of biocompatibility. Biomaterials. 2023;296: Article 122077. 36907003 10.1016/j.biomaterials.2023.122077

[20] Ren X, Zhang B, Guo G, Yu T, Yang L, Li G, Luo R, Wang Y. Research and prospects of strategies for surface-modified coatings on blood-contacting materials. Med-X. 2025;3(1):14.

[21] Fisher RA, Gollan B, Helaine S. Persistent bacterial infections and persister cells. Nat Rev Microbiol. 2017;15(8):453–464. 28529326 10.1038/nrmicro.2017.42

[22] Si L, Zhang S, Guo H, Luo W, Feng Y, Du X, Mou F, Ma H, Guan J. Swarming magnetic Fe_3_O_4_@polydopamine-tannic acid nanorobots: Integrating antibiotic-free superficial photothermal and deep chemical strategies for targeted bacterial elimination. Research. 2024;7:0438. 39086398 10.34133/research.0438 PMC11289052

[23] Foster TJ, Geoghegan JA, Ganesh VK, Höök M. Adhesion, invasion and evasion: The many functions of the surface proteins of Staphylococcus aureus. Nat Rev Microbiol. 2014;12(1):49–62. 24336184 10.1038/nrmicro3161 PMC5708296

[24] Zhu Y, Xu C, Zhang N, Ding X, Yu B, Xu F-J. Polycationic synergistic antibacterial agents with multiple functional components for efficient anti-infective therapy. Adv Funct Mater. 2018;28(14):1706709.

[25] Wu S, Xu C, Zhu Y, Zheng L, Zhang L, Hu Y, Yu B, Wang Y, Xu F-J. Biofilm-sensitive photodynamic nanoparticles for enhanced penetration and antibacterial efficiency. Adv Funct Mater. 2021;31(33):2103591.

[26] Lu Z, Zhang X, Zhao Y, Xue Y, Zhai T, Wu Z, Li C. BODIPY-based macromolecular photosensitizer with cation-enhanced antibacterial activity. Polym Chem. 2015;6(2):302–310.

[27] Zhou J, Zhang L, Wei Y, Wu Q, Mao K, Wang X, Cai J, Li X, Jiang Y. Photothermal iron-based riboflavin microneedles for the treatment of bacterial keratitis via ion therapy and immunomodulation. Adv Healthc Mater. 2024;13(26):2304448. 10.1002/adhm.202304448 39012057

[28] Zhao R, Zheng Y, Xu K, Huang L, Ding J, Shang X, Tao X, Xin S, Zheng Q, Qian Y, et al. A multistage microRNA nanotherapeutic to address fibrosis of bacterial keratitis. Nano Today. 2025;64: Article 102800.

[29] Peng Y, Pang S, Zeng Y, Wei J, Lu J, Ruan Y, Hong X, He X, Chu X, Guo Y, et al. Antibiotic-free ocular sterilization while suppressing immune response to protect corneal transparency in infectious keratitis treatment. J Control Release. 2024;374:563–576. 39186983 10.1016/j.jconrel.2024.08.038

[30] Li S, Lu Z, Huang Y, Wang Y, Jin Q, Shentu X, Ye J, Ji J, Yao K, Han H. Anti-oxidative and anti-inflammatory micelles: Break the dry eye vicious cycle. Adv Sci. 2022;9(17):2200435. 10.1002/advs.202200435 PMC9189644 35435328

[31] dos Santos GA, Ferreira-Nunes R, Dalmolin LF, dos Santos Ré AC, Anjos JLV, Mendanha SA, Aires CP, Lopez RFV, Cunha-Filho M, Gelfuso GM, et al. Besifloxacin liposomes with positively charged additives for an improved topical ocular delivery. Sci Rep. 2020;10(1):19285. 33159142 10.1038/s41598-020-76381-y PMC7648625

[32] Dorrington MG, Fraser IDC. NF-κB signaling in macrophages: Dynamics, crosstalk, and signal, integration. Front Immunol. 2019;10:705. 31024544 10.3389/fimmu.2019.00705 PMC6465568

[33] Morris G, Gevezova M, Sarafian V, Maes M. Redox regulation of the immune response. Cell Mol Immunol. 2022;19(10):1079–1101. 36056148 10.1038/s41423-022-00902-0 PMC9508259

[34] Li Y, Wang J, Li Y, Luo J, Liu F, Chen T, Ji Y, Yang H, Wang Z, Zhao Y. Attenuating uncontrolled inflammation by radical trapping chiral polymer micelles. ACS Nano. 2023;17(13):12127–12139. 37352508 10.1021/acsnano.2c12356

[35] Zhou X, Lu R, Jia M, Lai H, Xiao X, Yao Y, Yuan T, Li P, Zhang S. Degradation-dependent self-release hydrogel with over three months of antioxidation and anti-inflammation for osteoporotic bone repair. Chem Mater. 2024;36(7):3381–3394.

