# Supplementary material for: A “Detachable Polyanionic Protective Shell” Enveloping Photodynamic Cationic Nanoassemblies for Keratitis Treatment
Source: Research (Wash D C). 2026 Feb 25;9:1151. doi: 10.34133/research.1151 (PMC12935347; doi:10.34133/research.1151)
Supplement: Supplementary 1 — Figs. S1 to S13 Table S1 [file research.1151.f1.docx]

**A “Detachable Polyanionic Protective Shell” Enveloping Photodynamic Cationic Nanoassemblies** **for Keratitis Treatment**

Yuan Wei^1^, Yanyan Fu^2^, Haoyu Zou^3^, Yueze Hong^1^, Chak Kwong Cheng^4^, Meixia Zhang^2^, Rifang Luo^1^, Fanjun Zhang^1*^, Yunbing Wang^1*^.

1 National Engineering Research Center for Biomaterials, Sichuan University, Chengdu 610064, PR China.

2 Department of Ophthalmology, and Research Laboratory of Macular Disease, West China Hospital, Sichuan University, Chengdu, 610041, China.

3 Beijing Institute of Ophthalmology, Beijing TongRen Eye Center, Beijing Key Laboratory of Ophthalmology and Visual Sciences, Beijing Tongren Hospital, Capital Medical University, Beijing, China.

4 Department of Biomedical Sciences, City University of Hong Kong, Kowloon Tong, Hong Kong 999077, China.

* Corresponding author at: National Engineering Research Center for Biomaterials, Sichuan University, Chengdu 610064, China. E-mail address: [zhfanjun@163.com](mailto:zhfanjun@163.com); [yunbing.wang@scu.edu.cn](mailto:yunbing.wang@scu.edu.cn).

Figure S1. The ^1^H NMR spectra of PLL(Z)

Figure S2. The ^1^H NMR spectra of PLL


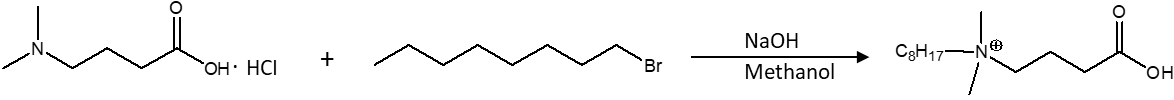


Figure S3. The synthesis process of QAS-COOH

Figure S4. The synthesis process and the ^1^H NMR spectra of QAS-COOH


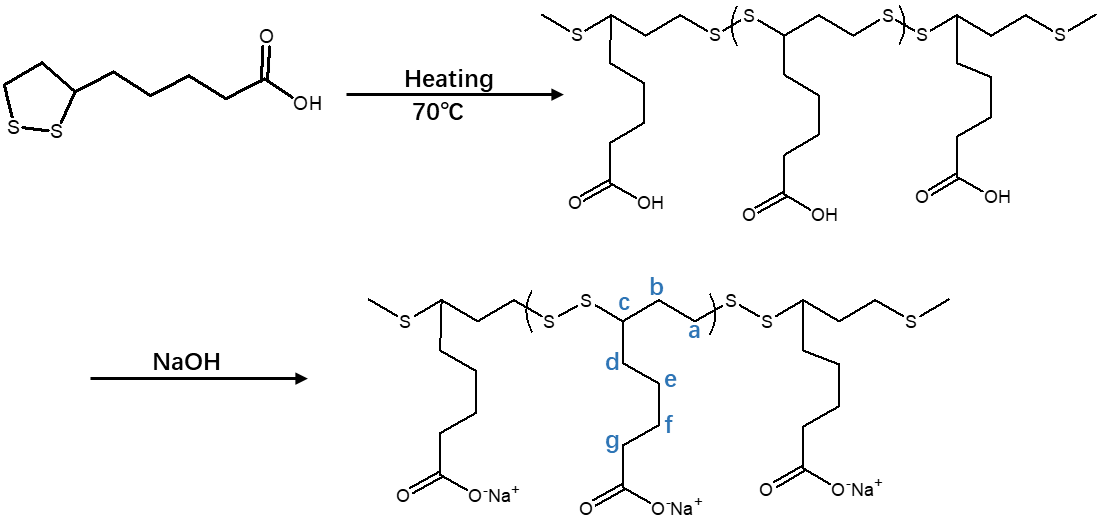


Figure S5. The synthesis process of PTA

Figure S6. The ^1^H NMR spectra of PTA

| sample | MIC (ug/ml) |
| --- | --- |
| PEG-PLL | 125 |
| QAS | 500 |
| PP-200%QAS | 125 |
| PP-400%QAS | 31.25 |
| PP-800%QAS | 62.5 |

Table S1. The minimum antibacterial concentration (MIC) of different samples


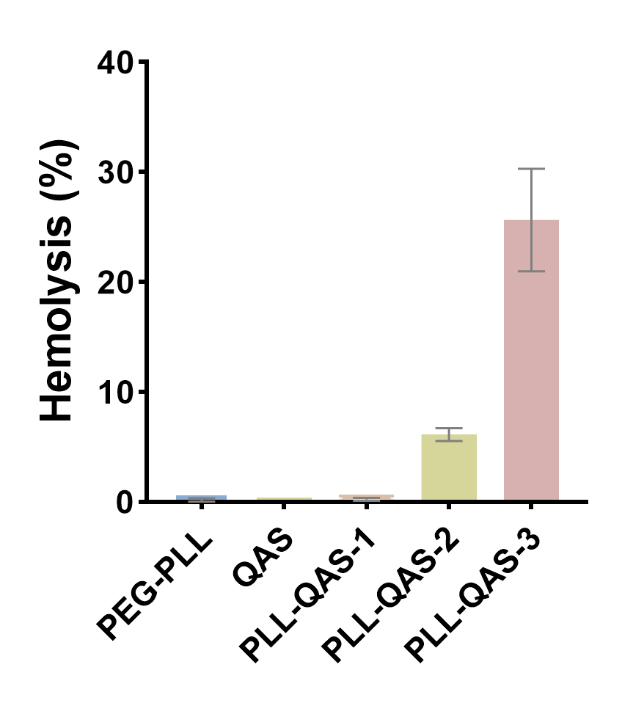


Figure S7. The hemolytic ratios of different samples


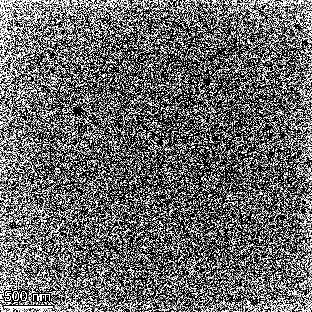

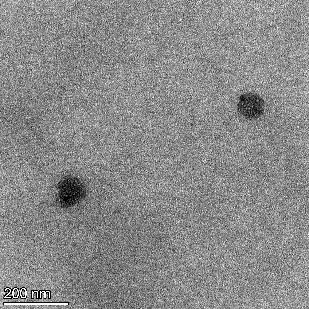


**200 nm**

Figure S8. TEM image of PQC nano-assemblies


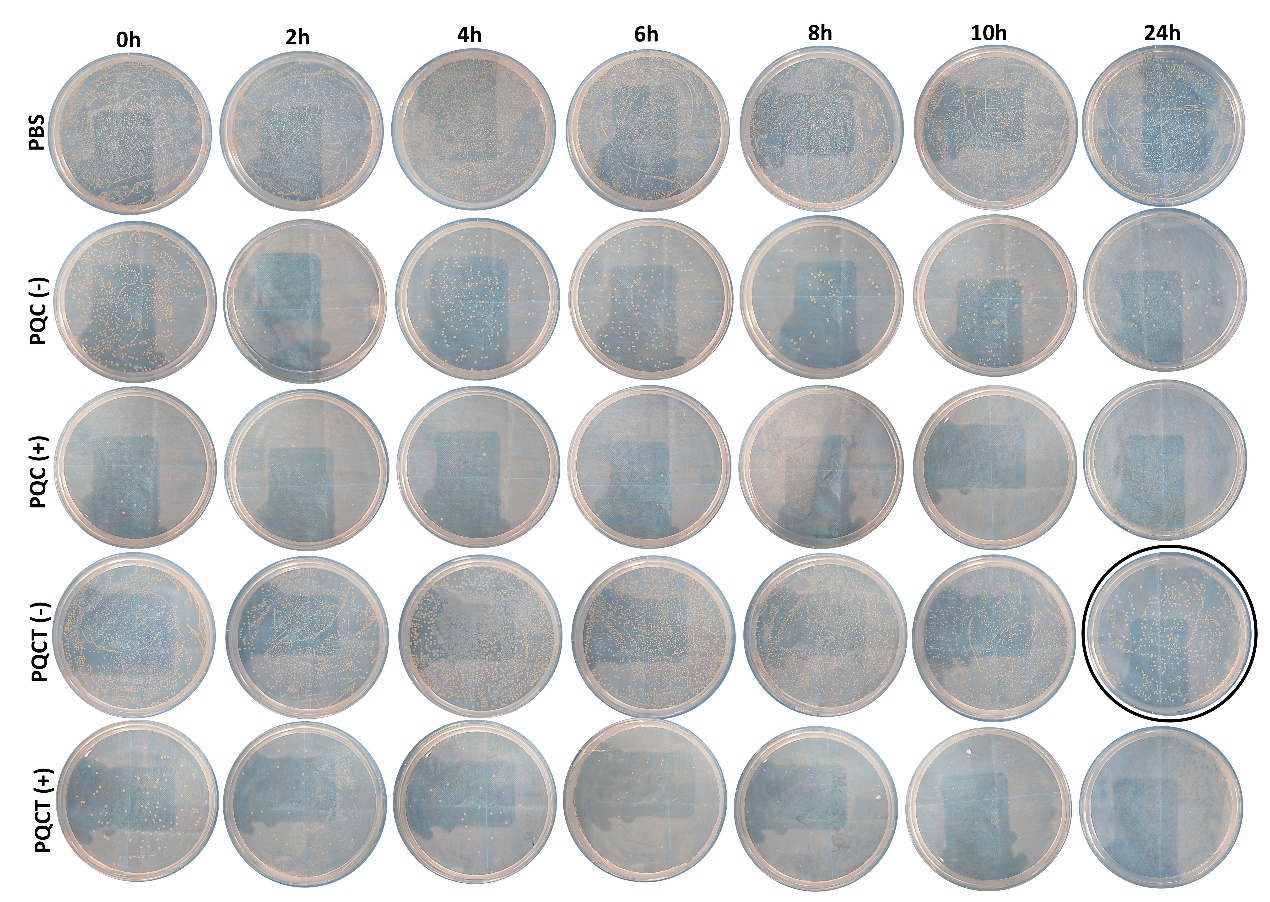


Figure S9. Visual view of an agar-coated plate. "(-)" means no irradiation treatment and "(+)" irradiation for 10 minutes (660nm,0.45W cm^− 2^)


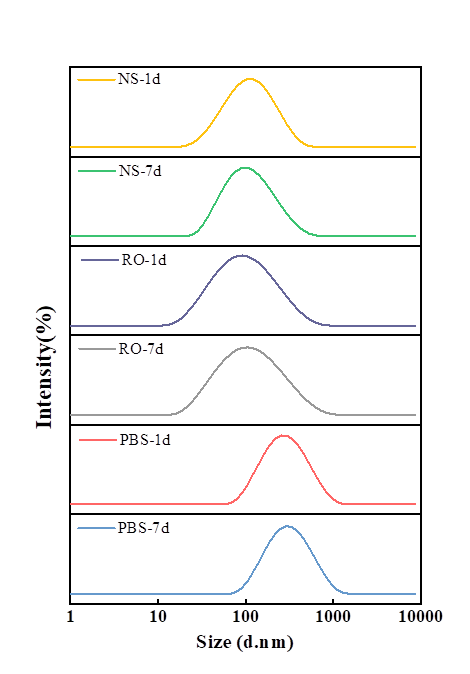


Figure S10. The size of PQCT in normal saline solution (NS) and deionized water (RO) and PBS solution on day 1 and day 7.

Figure S11. Cell viability was measured by CCK8 assay (n =6, mean ± SD), "(+)" denotes 10-minute irradiation (660 nm, 0.45W cm^−2^)


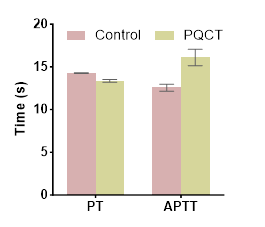


Figure S12. Detection of prothrombin time (PT), activated partial thromboplastin time (APTT) (n=3).


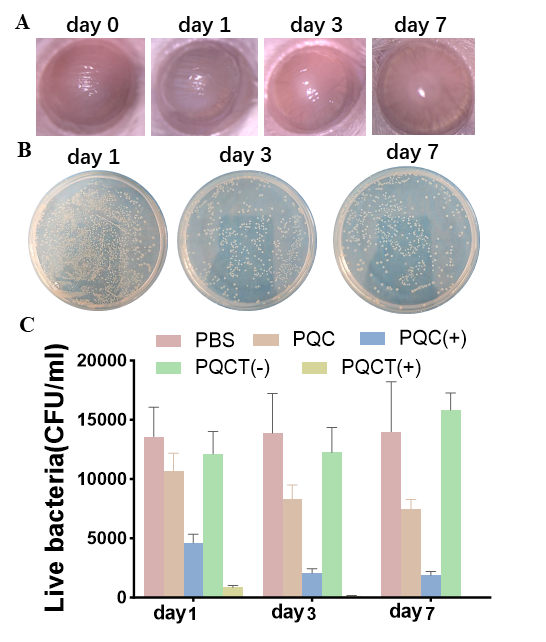


Figure S13. (A) Typical slit-lamp microscopy images of PQC (+) days 0, 1, 3, 5, and 7. (B) Bacterial colony visualization of PQC (+) on agar plates following tear fluid application at 1, 3, and 7-day intervals. (C) Matched numerical histograms illustrating bacterial counts in tear samples. The notation "(-)" indicates absence of light irradiation while "(+)" denotes 10-minute irradiation (660 nm, 0.45W cm^−2^).
